# Cost-Effective Implementation of a Temperature Traceability System Based on Smart RFID Tags and IoT Services

**DOI:** 10.3390/s20041163

**Published:** 2020-02-20

**Authors:** Oscar Urbano, Angel Perles, Cesar Pedraza, Susana Rubio-Arraez, María Luisa Castelló, María Dolores Ortola, Ricardo Mercado

**Affiliations:** 1 Grupo de Investigación EMC-UN, Universidad Nacional de Colombia, Ciudad Universitaria, 111321 Bogotá, Colombia; oaurbanov@unal.edu.co (O.U.); capedrazab@unal.edu.co (C.P.); 2 Instituto ITACA, Universitat Politècnica de València, Camino de Vera, s/n, 46022 Valencia, Spain; rmercado@itaca.upv.es; 3 Institute of Food Engineering for Development, Universitat Politècnica de València, Camino de Vera, s/n, 46022 Valencia, Spain; suruar@upvnet.upv.es (S.R.-A.); mcasgo@upvnet.upv.es (M.L.C.); mdortola@tal.upv.es (M.D.O.)

**Keywords:** traceability, Internet of Things, radio frequency identification (RFID) tags, wireless sensor network (WSN), cold chain, food monitoring for safety

## Abstract

This paper presents the design and validation of a traceability system, based on radio frequency identification (RFID) technology and Internet of Things (IoT) services, intended to address the interconnection and cost-implementation problems typical in traceability systems. The RFID layer integrates temperature sensors into RFID tags, to track and trace food conditions during transportation. The IoT paradigm makes it possible to connect multiple systems to the same platform, addressing interconnection problems between different technology providers. The cost-implementation issues are addressed following the Data as a Service (DaaS) billing scheme, where users pay for the data they consume and not the installed equipment, avoiding the big initial investment that these high-tech solutions commonly require. The developed system is validated in two case scenarios, one carried out in controlled laboratory conditions, monitoring chopped pumpkin. Another case, carried out in a real scenario, monitors oranges sent from Valencia, Spain to Cork, Ireland.

## 1. Introduction

The current global economy has changed the food industry by increasing the distance that food travels from producer to final consumer, bringing many challenges related to food traceability. Scandals such as genetically modified (GM) crops and foods, the outbreak of ‘chickengate’ in feedstuff, *Salmonella* and *Escherichia coli* in fresh fruit, or the horsemeat scandal in precooked food [1,2,3] have increased consumer concerns over the quality of food and the transparency about its origins. Keeping food quality and integrity during the whole supply chain prevents the outbreak of foodborne illnesses and even bioterrorism attacks, which are some of the major concerns [1].

Laws, policies, and standards have been developed attempting to regulate these issues. The International Organization for Standardization ISO 22005 Food Safety Standard attempts to ensure that there are no weak links in the supply chain, requiring that each company know their suppliers and customers. The ISO 22005:2007 gives codes and specifies the basic requirements for the design and implementation of a food and feed traceability system that can be applied by any organization operating at any step in the food chain. The traceability system is a technical instrument to assist an organization in conforming with its defined objectives and is applicable when necessary to determine the history or location of a product or its relevant components [4]. Within the traceability, two main concepts have to be considered: tracing and tracking. Tracing refers to determining the upstream path of the origin and characteristics of a product while tracking is the ability to follow the downstream path of a product along the supply chain. Then, traceability can be defined as a part of logistics management that captures, stores, and transmits adequate information about the product to all stages in the food supply chain so that stakeholders can check safety and quality control over their products [5,6].

In the European Union, the food law General Requirements are based on the safety of food because consumers must have confidence and assurance that the food they buy will not harm them or have an adverse effect. Thus, it establishes that only safe food and feed can be placed on the Union market or fed to food-producing animals. Besides, these requirements deal with the traceability of products throughout the food chain. The General Food Law Regulation defines traceability as the ability to trace and follow food, feed, and ingredients through all stages of production, processing, and distribution [3,7].

In the USA, the Food and Drug Administration (FDA) is adding regulations for the Current Good Manufacturing Practice, Hazard Analysis, and Risk Based Preventive Controls for Food for Animals. These regulations will, for the first time, establish requirements for the Current Good Manufacturing Practice (CGMP) for food for animals. In addition, the FDA is taking actions to provide greater assurance that animal food is safe and will not cause illness or injury to humans and animals. Nevertheless, the FDA Food Safety Modernization Act (FSMA) implements new rules intended to build an animal food safety system for the future that makes modern, science- and risk-based preventive controls the norm across all sectors of the animal food system [8].

Ensuring compliance of the traceability standards requires robust and reliable technological solutions able to track, trace, and notify about any problem along the supply chain, guaranteeing the safety of the food and consumer.

Emerging new technologies, such as smartphones, Internet of Things (IoT) or cloud computing, are changing rapidly the food industry. Access to the Internet through smartphones has made the food industry become more customer-oriented, as consumers can easily access product information. Customer complaints can also go viral through social networks, affecting a firm’s image. Regulations established by governments have forced companies to provide information about genetically modified (GM) products or its sources as customers are more aware of the origins and handling of their food [1]. To face all these issues, companies have implemented traceability systems based on diverse technologies, such as radio frequency identification (RFID), near field communication (NFC), deoxyribonucleic acid (DNA) barcoding, isotope analysis [9], or strips able to change based on temperature change [10]. Conceptual systems have also been proposed such as the TraceFood Framework [11] and the Food Track and Trace Ontology (FTTO) [12]. The challenge of these systems is the drive to overcome the economic barriers and to accomplish a successful technological transfer from tech companies to food companies or small producers, in order to take advantage of their huge potential.

Paper-based labeling systems are still being used by many small and big companies to keep control over their products. Barcodes are a cheap technology that provides identification and can be found within systems operating across the whole food chain. However, it does not provide any extra value, like automation or information storing. Many small companies in the food industry use this technology as they do not perceive any return on investment in high-tech systems that also require skilled personnel for maintaining [1,13].

Going beyond labeling and sensing systems, other techniques have been developed focusing the analysis of the chemical composition of the food. These techniques include stable isotope analysis, DNA barcoding, near infrared spectroscopy (NIRS), or chemometric methods (statistical modeling applied to analytical data to obtain chemical information). These techniques have proven to be enormously successful in detecting food origins when applied in conjunction [9]. However, most of them are too expensive to be applied in routine tests, but they can be useful in anti-counterfeit and verification scenarios.

RFID is a widely used technology in food logistics. This technology emerged in the 1950s and was made possible thanks to technological advances during the Second World War [14]. It consists of passive electronic labels placed on objects and RFID readers that gather information from the labels wirelessly. Due to its flexibility and scalability, it has been widely applied in logistics, inventorying, and many other industries [15]. In [16] the authors present an RFID-based system for tracking and tracing fresh vegetables in the supply chain, that allows the end consumer to know the complete history of the purchased food. In [17], RFID technology with embedded sensors also allows the development of temperature tracking systems during feeding logistics. Other authors [14,18] have also integrated RFID with several technologies (like wireless sensor network (WSN), IoT, or chemical-electrical sensors), making the most of its potential. The scope of the RFID technology was initially to provide wireless identification of assets, but thanks to its acceptance and development it has become the leading technology in the supply chain [13]. RFID has become a flexible solution suitable for many other different purposes such as blockchain implementation of traceability of wood [19], behavioral tracking of marine organism [20], cultural heritage monitoring [21], bee monitoring [22], or wearables [23]. At the same time, the read range of passive RFID tags is increasing while maintaining price, which is very interesting for the food supply chain or any type of logistics [24].

Near field communication (NFC) is another common technology in the food industry. NFC is a special case of RFID technology oriented to the near field exchange of information. It is widely used in payments and has been recently boosted thanks to the compatibility with some smartphones. This fact has turned NFC into a more customer-oriented technology. In the food industry, these labels are attached to products storing information relevant to the customer, and this data can be easily extracted using an NFC-compatible smartphone. Other authors [15] present a novel smartphone-based sensing strategy that implements chemiresponsive nanomaterials integrated into NFC tags able to measure gas-phase chemical concentration like ammonia, hydrogen peroxide, cyclohexanone, or water. NFC tags with temperature monitoring capabilities have also been implemented in medicine cold chain seeking to guarantee product quality and patient safety [25]. NFC technology has improved the potential of customer-oriented approaches by enabling the consumer to easily access traceability data about their food.

Wireless sensor networks (WSNs) are an active research line that counts many successful IoT applications [26,27,28,29]. It consists of the integration of different sensors connected through wireless technologies that implement standards such as ZigBee, XBee, 6lowPAN, or proprietary protocols [30]. These devices are far more complex than current RFID labels as they are designed for longer distances and the integration with many sensors toward non-centralized wireless communications. Some research trends in this area are reducing the power consumption or enabling ad-hoc or multi-hop communications (communications without the need of a central infrastructure) [31]. In traceability systems, WSN can be found in the physical layer, sensing and sending information about the conditions during the whole supply chain. Some authors combine WSN with RFID technology, which empowers the physical layer allowing asset identification and sensing information on the same network [18]. Although WSN and RFID were created with different purposes, advances in RFID have empowered this technology, and some authors even affirm that WSN and RFID are going to converge into one single technology, producing small, interconnected, and powerful devices [32], allowing an easy deployment of the Internet of Things.

Although these technologies have managed to solve many of the problems, there is still a lack of studies that show an effective implementation of these systems. Some of their strongest barriers are economic, as no return of investment is perceived, and interconnection problems, as different technology providers create different systems incompatible with each other. The goal of this paper is to show a practical implementation of a cost-effective traceability system, based on ISO 18000-6C RFID technology [33] with added sensing capabilities, that also allows the interconnection with different systems as it was developed following an IoT approach, making information accessible to all the stakeholders. The developed system is integrated and customized for two case studies, one simulating temperature conditions for chopped pumpkin and another one in a real case scenario in orange transportation.

## 2. Related Work

### 2.1. Internet of Things: A Game Changing Factor in Traceability

The Internet of Things (IoT) involves the integration and interaction between the physical/real and the digital/virtual worlds, where through the use of smart technologies (like wireless sensor networks, cloud computing, or artificial intelligence) the Things will be interconnected at any time, any place, and in any application [30]. Although it sounds like a futuristic paradigm, the IoT has been developed since the 1990s and has many successful application cases [25,27,29,34].

The current deployment of IoT relies on multiple technologies that can be applied depending on the application and its constraints. Some technologies like Wi-Fi and Bluetooth have been widely used for smart home applications taking advantage of the Internet network infrastructure already deployed in homes [30]. Some others such as Sigfox, LoRa or NB-IoT have been recently developed seeking low-consumption capabilities and massive deployment taking advantage of the cellular network infrastructure [35]. Other technologies are more industry-oriented, like RFID that has a leading role in the supply chain [36]. Some others are more customer-oriented, like NFC and its integration with smartphones that allows the consumer to access further information about the product before purchasing [9]. Thanks to these technologies, IoT is currently being deployed in multiple sectors, where the key is to find the technology that better fits into the requirements of the application.

In [37], a review of the current technologies implemented in IoT is shown. According to the authors, four characteristics must coexist in electronics developed for IoT: sensing capabilities, communication capabilities, energy autonomy, and eco-friendliness. Thanks to the recent advancements in RFID, such as sensing capabilities, energy harvesting and solutions to the security issues [37,38], RFID has the potential to fulfill these four aspects, and, according to [29], RFID and WSN technologies are going to converge, allowing small, cheap, and interconnected labels closing the gap between the objects, its data, and its digital representation [32].

### 2.2. Conceptual Framework

The conceptual framework has been mainly developed by the industry seeking to fulfill the standards and policies established by governments, dividing traceability into different entities, and focusing on the extracted valuable information more than the technology itself [9]. The Food Track and Trace Ontology (FTTO), developed in [12], is a relational model which presents a combination of four main classes (actors, food, process, and services) that can be implemented on the application layer (over multiple technologies), and, in case of any food outbreak, disease-related data can be extracted, obtaining essential information. The TraceFood Framework, developed as a collaboration of several EU-funded projects, is aimed to provide an international and non-proprietary standard for electronic data exchange between food traceability systems [11]. Also, the critical tracking events (CTE) approach has been considered. It changes the focus from being specific to food products to collecting data emphasizing more on the events that manipulate the products during the supply chain. The challenge of these conceptual approaches is to join them with the technology so all of their potential can be exploited [9].

### 2.3. Commercial Solutions

As the case study focuses on identification and cold-chain monitoring, a survey about the commercial solutions available for temperature monitoring was carried out. Table 1 presents a summary of the survey. Its purpose is to show the current state of the temperature monitoring solutions and the technologies these systems use. More than giving detailed description on the solutions, the purpose of this survey is to show the current state of the temperature monitoring solutions and the key points that will be improved by the proposed framework.

As can be seen in Table 1, typical commercial solutions for monitoring temperature (along with other variables) rely on complex devices, such as LoRa, SigFox, 4G, and Wi-Fi. Although these solutions implement leading technologies, most companies cannot afford them because companies look for more cost-effective solutions.

Although several approaches have been proposed to overcome interconnection and cost-implementation problems in traceability [11,16,39,40], IoT approaches could easily overcome these issues. Managing an IoT approach to the traceability cost-implementation problem could allow innovative concepts such as Data as a Service (DaaS) [41], overcoming the problem related to investment that small companies see when applying technology into their business core. With DaaS, clients pay for what they really care about, that is, the data related to their process, leaving technology handling to the tech companies in charge of maintaining the service. The IoT concept is also based on the sharing of information between different systems, and this characteristic can overcome the interconnection problems between different traceability systems. The Orbis Traceability System explained in the next section focuses on solving these two problems through the use of an IoT platform seeking to enable the interconnection between different systems and relying on cost-effective RFID labels with sensing capabilities. This develops a framework that can be applied in many traceability applications. As the data is stored in the cloud, any user-specific application can be built to consume this data, allowing the concept of DaaS to be exploited.

### 2.4. Orbis Traceability System

The implementation and operation of a traceability system is a complex and overwhelming task carried out by small companies whose business focus is food not technology. These small organizations mostly lack the financial capacity and trained personnel to implement it. Also, its implementation must comply with legislation and policies, guaranteeing food quality and information systems for all the stakeholders [6]. The development of these systems must rely on tech companies which also understand that the traceability problem is not the same for all companies as it involves different variables depending on the product.

The Orbis Traceability System is based on RFID technology with sensing capabilities because RFID is the leading technology in the supply chain [13] and one of the most promising technologies in IoT [28]. Its development is carried out following an IoT approach where the sensed data is stored in the cloud, available to all the stakeholders and third-party applications willing to use this information. As the system is validated through a cold-chain monitoring case, the data is gathered, processed, and presented in an on-line application developed for this case. 

**Figure 1:**
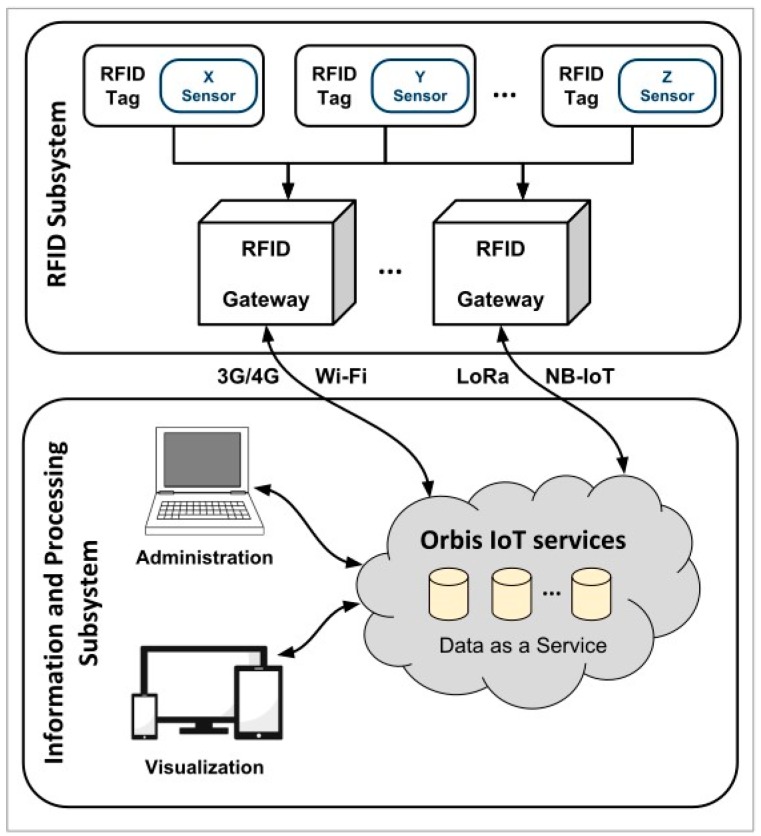
The architecture of the Orbis Traceability System.

The Orbis Traceability System is divided into two main subsystems: the RFID subsystem and the information and processing subsystem (Figure 1). The RFID subsystem implements RFID technology in the physical layer, querying information from the RFID tags provided with sensing capabilities, called smart-Tags. This data is sent to the cloud using gateways able to implement different telecommunication technologies, in this case RFID, Wi-Fi, and the mobile telephone network. The information and processing subsystem follows the concept of Data as a Service (DaaS) [41], where the stored data available on the cloud are then extracted and processed by an application that serves as a graphical user interface for all the stakeholders involved in the cold-chain monitoring case explained later.

**Figure 2:**
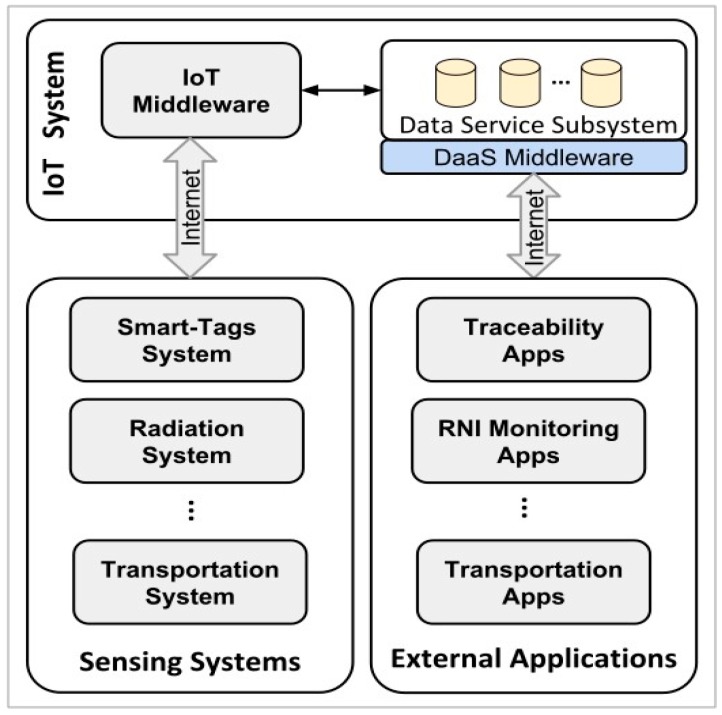
Internet of Things (IoT) Platform.

The IoT platform (Figure 2) is a cloud computing-based web platform, deployed on Google App Engine (GAE), taking advantage of its auto-scalability and high availability. It was initially developed in 2014 for the intelligent monitoring of public transportation by the National University of Colombia, obtaining public transport statistics and further information [42]. In 2016, in a project in conjunction with the National Agency for the Electromagnetic Spectrum Regulation, the platform was expanded and applied into the monitoring of non-ionizing radiation (NIR) levels in Colombia [43,44,45], intended for use as a means to regulate the use of the electromagnetic spectrum and its compliance. As a spin-off of this platform, Orbis Monitoring Solutions was created, which focuses on offering monitoring IoT solutions for third-party applications.

The cloud-based computing platform that uses RFID technology for the public transportation monitoring described in [42] is a patented product, achieved for the Electromagnetic Compatibility Group (EMC-UN), that now focuses its attentions on the supply chain, and toward the use of RFID technology, with sensing capabilities, proposing a cost-effective system to overcome integration and cost-implementation problems in traceability.

Google App Engine is a Platform as a Service (PaaS) that provides auto-scalability, so the system can be developed and tested at small scale, and when the massive deployment comes in, the system can grow flexibly, without the need of a redesign or performing any load balancing. PaaS is operating expenditures (OPEX) oriented, and its billing scheme is “pay as you go”, which means that the development phase is affordable and sometimes free, and during the operational phase the costs increase along with its use.

The IoT platform is composed of two main middleware subsystems, the IoT and DaaS middleware. The function of the IoT middleware is to handle all the incoming posts and queries from the sensing systems, while the DaaS middleware handles the sending of information to the external consulting applications. The scheme of DaaS is a key point for this system, as the billing of the whole IoT solution relies on it. The interaction with both middlewares is made possible by its application programming interface (API) that establishes standardized JavaScript Object Notation (JSON) forms for exchanging information. The DaaS middleware exchanges information with previously registered and authenticated applications that can process the data and provide further services, such as data mining for business intelligence or data analysis. These external apps are application specific and can be developed using any technology, platform, and language.

The main functionalities of the IoT platform can be summarized as:

- Receiving and verifying information sent by previously registered gateways and sensors, using Rivest, Shamir y Adleman (RSA) authentication keys.
- Providing services to manage (add, modify, and remove) data sources (sensors, gateways, and users).
- Interpreting, classifying, and storing incoming information.
- Ensuring privacy and integrity of the data as well as the auto-scalability of its databases, achieved through the use of the data storage model Google Datastore—BigTable.
- Avoiding Structured Query Language (SQL) injection attacks and denial of service attacks.
- Providing services to manage (add, modify, and remove) systems to use the services of the DaaS and IoT middleware.

## 3. Materials and Methods

### 3.1. RFID Subsystem

As described in Section 1, due to the standardization of RFID and its global adoption, the scalability it provides, and the advances in related technologies (that enable further capabilities in the tags), RFID has become the leading technology in the supply chain. It has also gained its place in many other fields, like transportation industries. In countries like Brazil, Mexico, Uruguay, and recently Colombia, this technology has been implemented in toll collection systems in roads, providing vehicle identification and enabling wireless fee payment [46]. RFID has an enormous potential and is definitely playing a key role in the deployment of the Internet of Things, which means that the research around this technology and its applications could lead to solving the interconnection and investment problems seen in traceability systems.

#### 3.1.1. Smart-Tags

RFID tags enhanced with sensing capabilities are not a new concept, and several implementations have been already carried out in many applications. In [47], RFID tags are combined with inkjet-printed sensors, enabling autonomous (solar-powered) soil moisture wireless sensors. In [19], this technology is combined with temperature sensors to develop tracking systems for feeding logistics. In [15], the authors study RFID systems for enabling wireless sensing in intelligent food logistics. The development of RFID tags enhanced with sensing capabilities is an active and innovative research line which leads to the development of more economical and powerful devices that can be used in traceability systems for gathering information about the conditions during food transportation.

**Figure 3:**
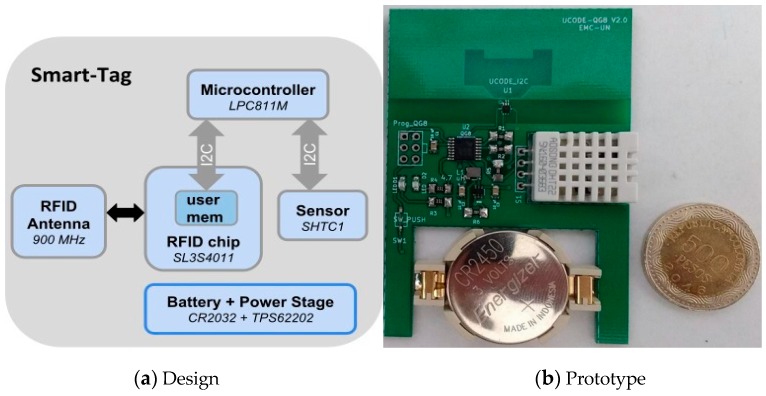
Smart-Tag.

Figure 3 presents the architecture of the smart-Tag developed for this project and its main hardware components. It is mainly composed of the RFID chip, a microcontroller, the specific sensor and the necessary electronics. This device was called smart-Tag because it goes beyond a simple wireless identification label, as it smartly saves sensing information into the user memory of the RFID chip. This means that any ISO 18000-6C compatible RFID reader can gather the information. However, due to its power-saving and economical features, it is a pretty constrained device that does not have much memory, so that the management of information is a key point for implementing this device successfully. Its development was made possible thanks to a versatile chip, provided by the NXP electronics company [48], that besides RFID implements Inter-Integrated Circuit (I2C) communications, allowing the integration of many sensors and electronics into the label. The microcontroller is a low-power device able to extract the information from the specific sensor (temperature and humidity sensor SHT1x from Sensirion). The battery and all the components were selected based on mass production criteria, seeking to achieve low-cost, reliability and scalability, enabling these devices to be integrated in any monitoring and tracking application, looking for small companies to finally invest in a cost-effective technological solution. Assuming that only temperature measurement is required, the mass production cost of this smart-Tag should be less than 2 Eur.

Typical data loggers for these applications often have several kilobytes of memory space. Nevertheless, the RFID chip just has 416 bytes available to the user, which is not enough for saving historical records during the cold-chain transportation, that often lasts weeks. However, if information about the timestamp on each sample is discarded, an account of the total time that the sensor was exposed to certain temperature values can fit, without any problems, into the small-Tag’s memory. Following this scheme, an effective method of sensing certain ranges can be implemented, storing into the RFID chip memory as much information as can be collected in one year. As will be seen in Section 4, following this method, no loss of critical information was produced, and the cold-chain conditions could be successfully interpreted. 

#### 3.1.2. RFID Gateway

**Figure 4:**
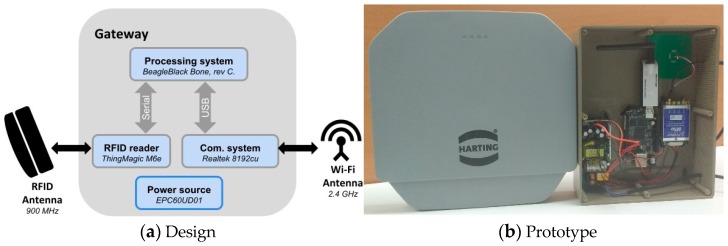
Gateway.

Figure 4 shows the architecture of the gateway and its main hardware components. The gateway implements RFID, Wi-Fi, and mobile network technologies to enable the communication between the smart-Tags and the IoT platform. It uses a commercial RFID reader for gathering the information from the labels, and through the processing system it extracts, encapsulates and sends the information to the IoT platform, making use of the available Internet network. The ThingMagic M6e RFID reader is a small and versatile device that enables the gateway communication with standard ISO 18000-6C RFID labels. It also has an open application programming interface (API) that abstracts all the low level reader protocol (LLRP) functions. This API is implemented in the processing system (BeagleBlack Bone) to query all the information from the RFID tags. The processing system executes an embedded distribution of Linux, which allows fast integration with many hardware communication subsystems used in IoT deployments such as, GPRS radio, LoRa devices, Wi-Fi, Bluetooth, etc. In this case, a Wi-Fi USB dongle device was used, taking advantage of the available wireless local area network.

### 3.2. Experimental Setup

Traceability systems are recognized as tools for guaranteeing food quality and safety, providing tracking and tracing along the whole supply chain, ensuring safer supplies and connecting producers and consumers in a transparent way [49]. Traceability has gained importance with the globalization of the food market, as food has to travel long distances, which increases the risk of cold-chain breakout or food contamination. These systems are based on technological solutions that comprise from simple printed labels to complex wireless sensor networks and information systems.

Advances in traceability systems can be divided into two approaches, the technology and the concepts. The technological approach focuses on the physical layer and through the implementation of leading technologies seeks to extract and control information about the product and its sources. On the other hand, the conceptual approach addresses the problem from the application layer, and it has been mainly developed by the industry seeking to fulfill the standards and policies established by governments, dividing traceability into different entities and focusing on the extracted valuable information more than the technology itself [9]. These two approaches complement each other, and many efforts have been carried out to fuse them into real scenarios [16,17,18,25,40,50]. 

This study presents the design and validation of a traceability system, based on RFID technology and IoT services, intended to address the interconnection and cost-implementation problems typical in traceability systems.

To validate the behavior of the whole system, we designed two case studies: local market pumpkin cold-chain monitoring and long-distance orange delivering.

**Figure 5:**
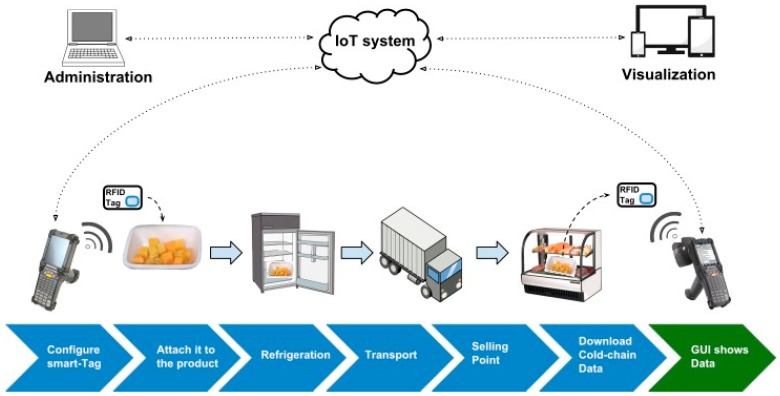
Experiment diagram.

Figure 5 explains the procedure of the experiments. The smart-Tag is attached to the product, following an initial scanning and configuration (temperature ranges, logging time, and electronic product code are set according to the application) performed by the RFID gateway that sends the initial time, location and configuration information to the IoT system. The smart-Tag will serve as a normal RFID label during the whole transportation and storing process, allowing any normal RFID traceability labor. Furthermore, it will be storing temperature information all along these processes. Once the box arrives to its destination, the information related to temperature can be downloaded using any ISO 18000-6C compatible commercial RFID reader.

#### 3.2.1. Pumpkin Cold-Chain Monitoring

Pumpkin (*Cucurbita moschata*) is a vegetable native to South Asia, and it belongs to the Cucurbitaceae family. It is a very important group of plants, not only from the economic point of view, but also due to its medical properties. In this sense, it has important compounds such as carotenoids with antioxidant properties [51].

Fresh pumpkin (*Cucurbita moschata, cacahuete* var.) was purchased in a local commercial market in Valencia, Spain. Fruits were selected for their uniformity in shape, weight and color in autumnal harvest. In this experiment, a pumpkin (weight: 1.5 kg, caliber: 150/200 mm, origin: Toledo (Spain)) was cut into cubes, and then 150 g of pumpkin cubes were placed on four plastic trays and then sealed. The white plastic trays (PP, mod. 5050, ATS PACKAGING s.r.l., Venice, Italy) and the film (Toplex HB 37, ILPRA, Barcelona, Spain) used to seal the trays are compatible with food packaging. The sealing conditions were 165 °C for 3.5 s with compressed air without vacuum. The experiment was carried out in collaboration with the Institute of Food Engineering for Development (IUIAD) of the Universitat Politècnica de València, specifically, in the Fruit and Vegetable Postharvest and Conservation Laboratory.

Four stages were simulated in this investigation, as can be seen in Table 2, (refrigerated chamber, transport, selling point, consumer home). In this case, two plastic trays with pumpkin cubes were monitored with the Orbis Traceability System. In the first stage, the trays with pumpkin cubes were kept in a refrigerated chamber at 4 °C for 7 days to simulate the postharvest, processing and packaging stages. In the second stage, the pumpkin cubes were transported to the point of sale. During the transport, they remained at 7 °C for 3 h and at 4 °C for 21 h to preserve the food cold chain. In the third stage, samples were placed in conditions to simulate the selling point, at 4 °C for 7 days. Lastly, in the fourth stage, pumpkin cubes were submitted to domestic conditions of refrigeration (7 °C) for 7 days.

Previous studies carried out with dehydrated pumpkins to compare the kinetics of carotenoid degradation depending on temperature and atmosphere revealed that the recommended condition for these products is N_2_ atmosphere at 4 °C. Therefore, package atmosphere is fundamental during long-term storage [52].

#### 3.2.2. Orange Temperature Monitoring

On the other hand, the orange is also a fruit native to Asia. Citrus fruits such as orange (*Citrus sinensis*) belong to the *Rutaceae* family, and they have many beneficial properties due to their high content of vitamins, fiber, minerals, ascorbic acid and very high content of antioxidant compounds, such as flavonoids, carotenoids and phenolic compounds [53].

**Figure 6:**
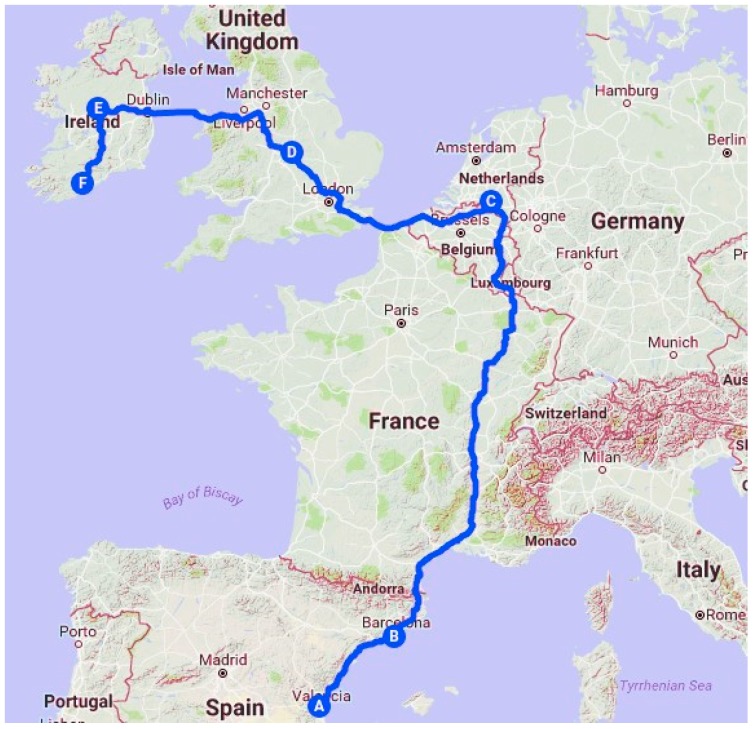
Orange tracking.

The second case study was carried out by monitoring the temperature of a box of oranges sent from Valencia (Spain) to Cork (Ireland). Figure 6 shows the route traveled by this product, which lasted 8.8 days.

Fresh oranges (*Citrus sinensis*, Navelate var.) were provided by Naranjas de Cullera, S.L., a small company specialized in direct harvesting and delivery to customers. Fruits were selected for their uniformity in shape, weight and color in winter harvest. In this experiment, the oranges presented Category I, caliber: 6 (60/80 mm/unit), total weight: 7 kg, origin: Valencia (Spain). They were boxed in cardboard for transport during the late winter without specific cooling conditions, only the typical winter environmental conditions. Fruits should not be frozen, and the recommended temperatures for storage are in the range of 2 to 8 °C [54].

The experimental procedure is the same as explained in Section 3.2.1, but this time the experiment was carried out in a real scenario outside from the laboratory.

This study proposes a food traceability system, utilizing RFID technology to track and trace product temperature and humidity during storage and transportation. The proposed food traceability system will help not only to optimize food distribution but also to increase customer satisfaction, since product freshness is monitored.

According to this experiment, Table 3 shows the possible ranges of temperature values for oranges in the different stages of the distribution. Therefore, it is very important to keep the temperature ranges between 2 and 8 °C, to maintain the cold food chain. However, in this case, fruits like oranges have a hard skin that protects them from changes in temperature and presents a greater resistance during the transport process to be a whole fruit. However, it is possible to detect damages by cold (in the case of excessively low temperatures) or damages by the action of microorganisms (molds) that could have affected the surface of the product due to blows or breaks of the packaging.

## 4. Results and Discussion

**Figure 7:**
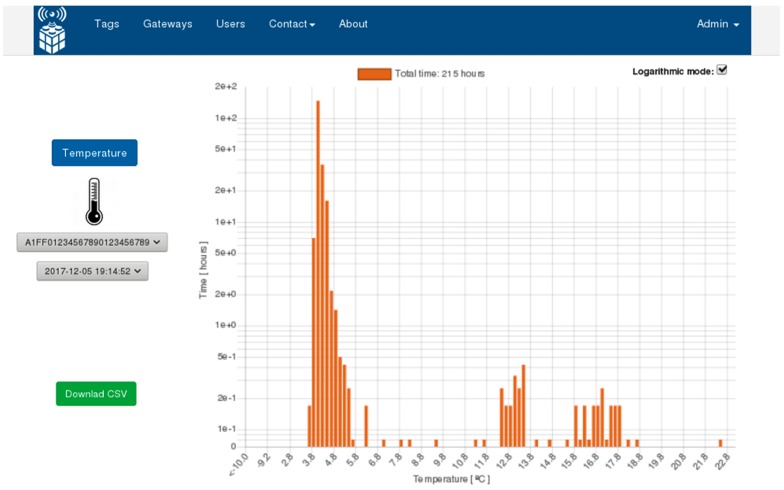
Graphical user interface, third-party application.

The extracted information is processed by the gateway and sent to the IoT system, registering arrival time and storing conditions. These data are stored in the cloud where through the DaaS middleware it can be accessed at any time. In this case, a third-party application was designed (Figure 7) in order to show times and temperature-storing information during the four stages, where the data are also available for downloading in comma-separated values (CSV) format for further custom analysis. To validate the temperature monitoring operation, we added a calibrated Testo 174H data logger in all of the experiment phases.

To validate the temperature monitoring operation, we added a calibrated Testo 174H data logger able to record temperature and humidity in all of the experiment phases. However, for practical reasons only temperature is considered in this experiment. The ability to integrate sensors from different providers, was one of the problems (interconnection problems) to overcome with the design of this framework, it shows the versatility of the IoT system, being able to store information from different types of systems. In the case of the Testo data logger information, it was obtained using a CSV format and, through the IoT middleware, the data was transformed and sent to the IoT platform.

### 4.1. Pumpkin Cold-Chain Monitoring

**Figure 8:**
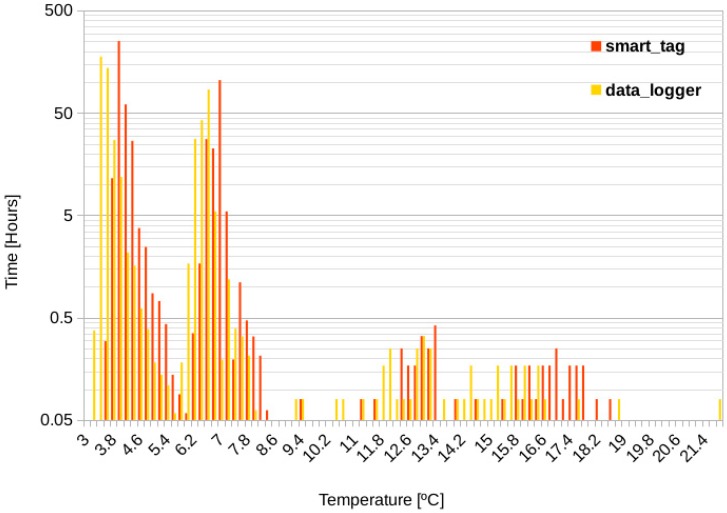
Cold monitoring case.

Figure 8, with the Y axis in logarithmic scale, shows the results of both, measurements recorded by the datalogger and measurements extracted from the smart-Tag. Every bar in the graph represents the time that the devices have been exposed to a given temperature range. Although measurements in both devices are distributed following the same pattern, differences can be appreciated in the peaks, typically lower than ±0.5 °C, which is in fact the accuracy guaranteed for both sensors in its datasheets. 

From this graph, information regarding temperature monitoring and therefore conditions during the food cold chain can be extracted. Table 4 summarizes the amount of time the smart-Tag was exposed to different temperature ranges and its interpretation.

Regarding pumpkins temperature ramps (Table 2), it is needed to keep the temperature values between 4 and 7 °C, in order to preserve the cold chain for this product. As can be deduced from Table 4, the temperature ranges were respected during the chamber and selling point. However, during 4 h the product was out of the allowed ranges, which corresponds with the manipulation time during the transportation, 3 h of simulated normal transporting conditions, where the product was deposited in a normal extruded polystyrene thermal box which did not keep optimal temperature conditions. 

### 4.2. Orange Temperature Monitoring

**Figure 9:**
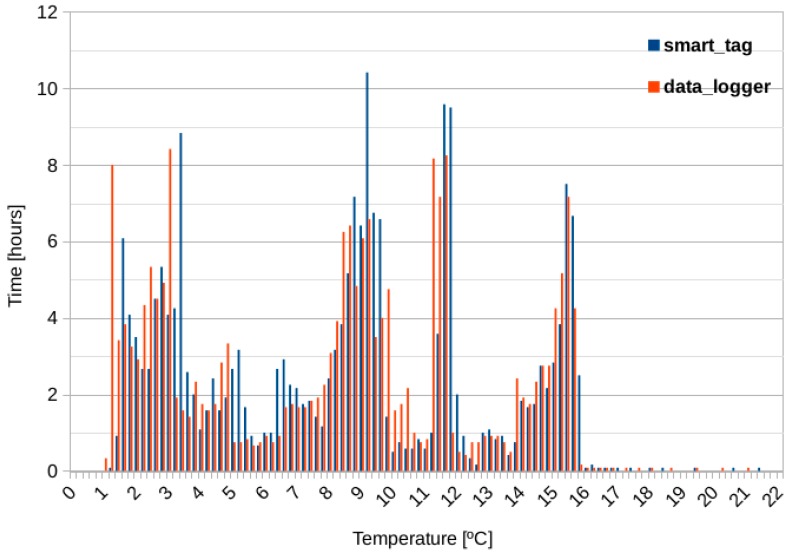
Orange temperature monitoring.

Figure 9 shows the results for the orange case monitoring. As for Figure 8, measurements follow the same distribution for both devices, with maximum differences typically lower than ±0.5 °C appreciated in the peaks.

Table 5 shows the interpretation of results extracted from the above figure, it summarizes the amount of time the smart-Tag was exposed to different temperature ranges and its interpretation.

Considering this table we can conclude that, for 3.68 days the oranges were kept at good temperature conditions. But, during 4.25 days, there was an exposure to a range (9–22 °C) higher than the suitable. However, even though this temperature was not optimal the product arrived in good conditions to its destination, thanks to the roughness of its skin, oranges can support a variety of climate conditions typical to its transport process. Moreover, the lowest temperature registered was 1 °C, and the lower breakpoint was never reached (below −5 °C), so no cold chain break was detected for this product.

## 5. Conclusions and Future Work

The IoT platform solves the integration problems by receiving data from different providers and making it available to different customers.

In this case, data was obtained by using two different technologies: the smart-Tag system and the Testo data logger system. In the case of the smart-Tags, the application that translates data into the format received by the IoT platform is already integrated into the RFID gateway. In the case of the Testo data logger, it is a standalone product, that uses a Windows application and, with a USB interface, stored data can be downloaded in a CSV file format. This data were processed by an application developed in Python (along the IoT middleware), able to read all the data from the CVS and transform it into the JSON format accepted by the IoT platform. Once data is on the cloud, it is consulted by the external application shown in Figure 5 using the DaaS middleware. It is a web application developed using standard technologies (HTML, PHP7, and JavaScript) and shows further information obtained from the smart-Tag system. The web application was created for the cold-chain monitoring case and shows information about temperature and humidity related to each smart-Tag as well as user management. Moreover, standard RFID tracking applications can be involved.

Compared with typical temperature monitoring solutions, which use robust and expensive wireless hardware, as it takes advantage of the scalability and low prices point of the RFID technology. As the ones described in Section 3.1.1, the RFID smart-Tags proved to be cost-effective, and the whole system is scalable and suitable for the cold-chain monitoring application presented in this paper. RFID was considered over multiple technologies, due to its economy, maturity, and adoption by the industry, as it is present in the whole supply chain. Furthermore, the DaaS scheme opens a lot of new possibilities for tech providers and tech consumers, as it transforms billing schemes into a more affordable model (especially for small food companies), where initial investment price is highly decreased. Moreover, RFID technology and cloud computing constitute scalable solutions, since RFID was made for automatic identification and can handle simultaneous communication with multiple tags, while cloud computing allows automatically growing databases and information services, without worrying about the physical infrastructure.

Assuming that only temperature measurement is required, the mass production cost of this smart-Tag should be less than 2 Eur. If we consider the commercial margins, this type of solution would be suitable for those goods where the balance between the cost of the intelligent labelling and the product is adequate.

In the fruit and vegetable market, consumer safety has become one of the most critical and priority issues in the food chain. Despite the efforts from members of the food chain for fresh products, food security problems cannot be eliminated easily. Vegetables, fruits, and food in general, require a comprehensive, integrated, and effective control system to warranty good conditions during the whole food chain. Therefore, an effective and cost-efficient traceability system can significantly improve tracking accuracy and speed access to information about food production and destination. The RFID technology used by the developed framework allows wireless immediate identification, without vision line with the reader, since the information is transmitted by radio frequency waves. This technology enables to do the traceability of the products while at the same time allows to save extra data in the RFID tags memory. Therefore, it brings great value to the consumer while guaranteeing the good conditions on the products, since a large part of the nutritional and vitamin characteristics will depend on the time that passes from its collection to its consumption. From a public health perspective, by improving the speed and accuracy of the tracking and location of the food items involved, it can help reduce risks related to food safety. Moreover, the tracking of food items can help public health services and industrial operators to determine the potential causes of a problem with integrated traceability from the origin, thereby providing the necessary individual information to identify and minimize health risks [55]. 

With the data extracted by the traceability system described in this paper, and some information regarding logistics of the products, it was possible to reconstruct the cold chain and detect the compliance of temperature conditions. However, it was not possible to obtain information about the time when the cold chain break actually occurred, nor its recurrence. Due to the low-memory typically available in RFID chips, information regarding timestamps had to be sacrificed, but if we consider that the monitoring of most of the food-products should comply just with certain ranges, we could manage a different schema for mapping the events in memory, giving more details on timestamps and less granularity for the measured values. Therefore, as future work and an evolution of this framework, it will be considered customizable memory-mapping schemas depending on the product to monitor, which will allow us to record timestamps and be able to reconstruct the cold chain break events including timestamps, at the expense of less granularity in the measures, which at the end is not critical for this application. Also, in case of long periods for a cold chain break, the smart-Tag could emit a visual or audible signal, in order to warn consumers on the state of the product.

## Figures and Tables

**Table 1:** Temperature tracking commercial solutions.

Product	Description	Technology
SensoTag NFC	Range: −40 to 60 °C Records: 4000	Protocol: NFC Interface: Smartphone App, Cloud
EMERALD Temperature Recorder	Range: −40 to 85 °C Records: 4000	Protocol: Bluetooth Interface: Smartphone App, Cloud
Cobalt ML3 Temperature Recorder	Range: −40 to 60 °C Records: 4000	Protocol: LoRaWAN Interface: Smartphone App, Cloud
Monnit Wireless Temperature Sensor	Range: −7 to 60 °C Records: 512	Protocol: Not Available (900 MHz) Interface: RF 900 MHz, Cloud
CCP Smart Tags	Range: Not Available Monitoring: Critical Points	Protocols: Wi-Fi, NFC, 4G, Sigfox, NBIoT Interface: Cloud
StickNTrack TEMP GPS	Range: −20 to 60 °C Monitoring: GPS and Temperature	Protocol: Sigfox Interface: Cloud

**Table 2:** Pumpkin temperature ramps.

Stages	Temperature (°C)	Time (days)
Chamber	4	7
Transport	7 (3 h), 4 (21 h)	1
Selling Point	4	7
Consumer Home	7	7

**Table 3:** Orange temperature classification.

Description	Temperature Range (°C)
Above the optimal temperature	9–22
Optimal temperature	2–8
Low temperature break point	<−5

**Table 4:** Pumpkin time range interpretation.

Interpretation	Temperature Range (°C)	Time (days)
Chamber and selling point	3.2–5.2	14.96 days
Transport	5.8–8.4	6.86 days
Manipulation time	9–22	3.99 h

**Table 5:** Orange condition interpretation.

Interpretation	Temperature Range (°C)	Time (days)
Above the optimal temperature	9–22	4.25
Refrigerator temperature	6-8	0.76
Refrigerator temperature	1–6	2.92
Low temperature break point	<−5	0
